# Evaluating the Effectiveness of a Local Primary Care Incentive Scheme: A Difference-in-Differences Study

**DOI:** 10.1177/10775587211035280

**Published:** 2021-07-29

**Authors:** Esmaeil Khedmati Morasae, Tanith C. Rose, Mark Gabbay, Laura Buckels, Colette Morris, Sharon Poll, Mark Goodall, Rob Barnett, Ben Barr

**Affiliations:** 1 University of Exeter Business School, Exeter, UK; 2 University of Liverpool, Liverpool, UK; 3 Liverpool Clinical Commissioning Group, Liverpool, UK; 4 Liverpool Local Medical Committee, Liverpool, UK

**Keywords:** emergency admission, quality improvement scheme, incentive, general practice

## Abstract

National financial incentive schemes for improving the quality of primary care have come under criticism in the United Kingdom, leading to calls for localized alternatives. This study investigated whether a local general practice incentive-based quality improvement scheme launched in 2011 in a city in the North West of England was associated with a reduction in all-cause emergency hospital admissions. Difference-in-differences analysis was used to compare the change in emergency admission rates in the intervention city, to the change in a matched comparison population. Emergency admissions rates fell by 19 per 1,000 people in the years following the intervention (95% confidence interval [17, 21]) in the intervention city, relative to the comparison population. This effect was greater among more disadvantaged populations, narrowing socioeconomic inequalities in emergency admissions. The findings suggest that similar approaches could be an effective component of strategies to reduce unplanned hospital admissions elsewhere.

## Introduction

The United Kingdom has been at the forefront of developing incentive schemes to improve the quality and efficiency of general practice. The Quality and Outcomes Framework (QOF) was introduced nationally in the United Kingdom in 2004, allocating 25% of general practitioners (GP) income based on the achievement of quality targets for the management of chronic and severe conditions, as well as indicators related to practice organization and patient experience (Guthrie et al., 2006; Roland & Guthrie, 2016; see https://qof.digital.nhs.uk/ for a description of the national indicators).

The QOF has, however, received increasing criticism with several studies suggesting it has not led to improvements in the quality of primary care and population health metrics, and that it is poor value for money (Ashworth & Gulliford, 2017; Campbell et al., 2009; Doran et al., 2006; Doran et al., 2011; Doran et al., 2014; Doran et al., 2017; Fleetcroft et al., 2010; Forbes et al., 2017; Gillam et al., 2012; Grigoroglou et al., 2018; Hamel et al., 2014; Kontopantelis et al., 2015; Lester et al., 2010; Mandavia et al., 2017; Marshall & Roland, 2017; Raleigh, 2018; Roland, 2016; Roland & Guthrie, 2016; Roland & Olesen, 2016; Ryan et al., 2016; Steel & Shekelle, 2016; Thorne, 2016). There have been a considerable number of studies and systematic reviews investigating the effects of incentive schemes on quality, cost, efficiency, and equity of primary care provision (Alshamsan et al., 2010; Alshamsan et al., 2012; Conrad & Perry, 2009; Damberg et al., 2014; Doran et al., 2006; Eijkenaar et al., 2013; Emmert et al., 2012; Flodgren et al., 2011; Girault et al., 2017; Glidewell et al., 2015; Grimaldi, 2018; Herbst et al., 2018; Houle et al., 2012; Kondo et al., 2016; Lee et al., 2011; Markovitz & Ryan, 2017; Mendelson et al., 2017; Pandya et al., 2018; Petersen et al., 2006; Petersen et al., 2013; Roland & Dudley, 2015; Scott et al., 2011; Soucat et al., 2017; Van Herck et al., 2010; Walker et al., 2010; Witter et al., 2012; Yuan et al., 2017), often with inconsistent results (Bardach et al., 2013; Emmert et al., 2012; Houle et al., 2012; Roland & Dudley, 2015). Whilst there is some evidence that they can improve process measures, there is a lack of evidence for improvements in patient health indicators (Flodgren et al., 2011). Some experimental economic studies have, however, suggested that such schemes may lead to more optimal use of resources (Green 2014; Hennig-Schmidt et al., 2011; Brosig-Koch et al., 2016; Xi et al., 2019). Studies investigating the health inequalities impact of such schemes have been also mixed (Alshamsan et al., 2010), with some studies indicating that they increase inequalities (Alshamsan et al., 2010; Roland & Dudley, 2015), whilst Doran et al. (2008) showed that QOF was successful in reducing the inequalities indicators for the first 3 years of deployment, though it was not persistent in its positive effects (Dixon & Khachatryan, 2010). Due to this mounting criticism, there have been suggestions to amend the QOF (Doran et al., 2017; Minchin et al., 2018; Pandya et al., 2018), including handing control over to local primary care systems (Hackett et al., 2014; Glidewell et al., 2015). There is, however, a lack of evidence available to inform local incentive and quality improvement schemes (Hackett et al., 2014).

We therefore aimed to investigate the effect on all-cause emergency admissions of a local primary care quality improvement scheme implemented in 92 GP practices in a northern U.K. city (see The Intervention section for further detail and a conceptual framework). Using data on emergency admission rates for small neighborhoods, we investigated the change in rates before and after the intervention in the study site and in a set of matched comparator areas.

### New Contributions

Incentive schemes have been viewed as valued ways of funding primary care services amidst overall reductions in primary care financing. Some evidence suggests that national incentive schemes in the United Kingdom have had limited success in improving patient outcomes, for example, reductions in unplanned hospitalizations. This has prompted calls for localized alternatives to capture the specificities of local contexts. Evaluation evidence of localized incentive schemes is lacking, especially in relation to effects on health inequalities. We therefore evaluated a local incentive scheme developed and scaled up in a city in the North West of England. This local scheme was implemented in addition to the nationwide incentive scheme and involved four key components: (1) an increase in the level of funding for general practices, (2) distribution of that funding proportional to the level of need in the populations served by each practice, (3) a set of locally tailored performance targets, and (4) a governance framework overseen by the GPs themselves. The findings suggest that these components were effective in reducing the level of and inequalities in all-cause emergency hospitalizations. Quality improvement incentive schemes, including these components may be effective in other settings.

## Method

### Setting

The intervention was implemented across a city in North West of England, with a population of 550,000. It is the fourth most deprived local authority in England based on the Indices of Multiple Deprivation (Department for Communities and Local Government 2015). Healthy life expectancy is only 58 years—significantly lower than the 63 years nationally. The intervention included all 92 GP practices serving this population.

### Study Design

This study was a longitudinal matched controlled study using difference-in-differences analysis. This difference-in-differences method controls for all time-invariant differences between the intervention and control populations (supplementary file, Appendix 1, available online). The key assumption of difference-in-differences analysis is the parallel trends assumption. If the trend in the outcome in the intervention and control populations would have been parallel in the absence of the intervention, then the difference between the change in the outcomes and between the two groups provides an unbiased estimate of the interventions effect (Dimick & Ryan, 2014).

Lower Layer Super Output Area (LSOA) years were the units of analysis. LSOAs are small geographical areas used by the UK’s Office for National Statistics (ONS), each typically containing a population of about 1,500 people. England is divided into just over 30,000 LSOAs. Two hundred and ninety eight LSOAs cover the entire population of the intervention city. Each of these intervention LSOAs was matched with five control LSOAs located within other districts in the North West region of England, providing 1,490 matched control LSOAs—that is, 1,788 LSOAs in total. In order to identify a matched control group that was likely to satisfy the parallel trends assumption, we used propensity score matching (Rosenbaum & Rubin, 1983) to identify control areas that had experienced similar trends in emergency admissions and predictors of this outcome (age, unemployment, proportion of the population that were female) in the time period before the introduction of the intervention (2005-2010; see online supplementary file, Appendix 2, for full details of the matching variables). These three variables were included in the matching and as time varying controls as they include the main factors that predict health care utilization (i.e., age, sex, and socioeconomic deprivation), and they were available annually for LSOAs (see The Intervention section). Other morbidity measures were considered (e.g., prevalence of chronic conditions), however, these would have introduced bias as the diagnosed prevalence of these conditions could have been affected by the intervention itself through increased access to diagnosis.

We then used a linear regression model to compare the change (difference) in the emergency admission rate in the intervention population with the change (difference) in the emergency admission rate in the matched comparison population, 6 years before (2005-2010) and 6 years after (2011-2016) implementation. We calculated emergency hospital admissions per 1,000 population for each of the 1,788 LSOAs between 2005 and 2016, giving a total sample size of 21,456 LSOA-years. To adjust for time varying factors that could be associated with trends in emergency admission rates, we controlled for trends in the annual average age of the population, the percent female, and the unemployment rate measured as the proportion of the working age population (aged 16-64 years) claiming unemployment benefits. We investigated the parallel trends assumption using graphical methods and regression models to compare trends in emergency admission rates between the intervention and control populations in the preintervention period (2005-2010). To investigate whether there was a difference in effect in more compared with less deprived areas, we conducted subgroup analysis across groups defined by their level of income deprivation. Parallel trends were investigated for each subgroup.

To test the sensitivity of the analysis to the control group chosen, we repeated the analysis using GP practice—years as the unit of analysis and using controls selected from outside the North West region of England. Additionally as the process of matching can introduce bias related to regression to the mean (Daw & Hatfield, 2018), we conducted supplementary analysis using the synthetic control method for microdata (Robbins & Davenport, 2021). This uses a weighted combination of all of the available “untreated” LSOAs, instead of just a matched sample (see online supplementary file, Appendix 6).

All intervention practices were included in this analysis even if their funding was withdrawn, due to under performance (see below). This intention to treat analysis will provide a more conservative estimate of the intervention effect and is less prone to bias.

### The Intervention

**Figure 1.**
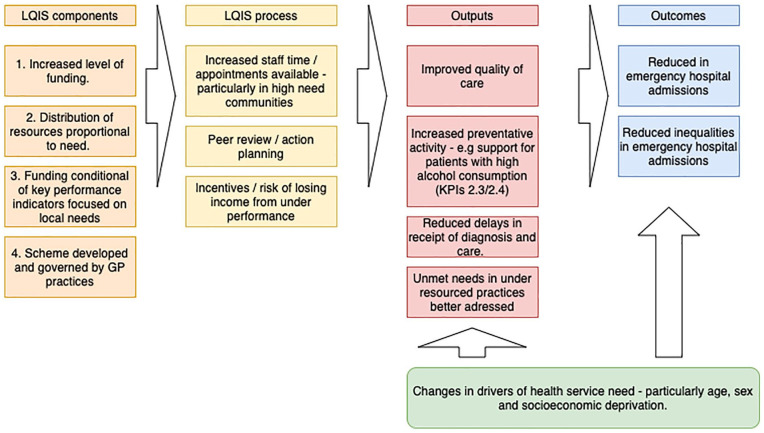
Conceptual framework showing input components and hypothesized process and outputs leading to the outcomes. *Note*. LQIS = Local Quality Improvement Scheme; GP = general practitioner.

The Local Quality Improvement Scheme (LQIS) has four main components as outlined in the conceptual framework in Figure 1.

First, there was an increase in the level of funding. An additional £5 million a year, £30 million in total was invested in the GP practices between 2011 and 2016 through this scheme. Second, these funds were distributed proportional to the level of need. Prior to the intervention, idiosyncrasies in national contracting arrangements and differences in the uptake of optional enhanced services had led to considerable variation in the funding each practice received ranging from £51 to £207 per population, weighted for need using the Carr-Hill Formula. The Carr-Hill formula is used in the United Kingdom to adjust populations for their relative need for primary care—taking into account the age and sex of the population and the standardized prevalence of limited long-standing illness and the standardized mortality ratio for patients younger than 65 years (British Medical Association, 2020). The LQIS equalized these payments ensuring that each practice received a minimum of £90 per needs weighted population. Third, receipt of this additional funding was conditional on achieving a set of 13 key performance indicators (KPIs) that were monitored on an annual basis (see online supplementary file, Appendix 3, for a list of KPIs). These indicators were developed in collaboration with local GPs and public health specialists. They were designed to address specific health needs relevant to the local population. In particular, this involved increasing primary care access and early identification of chronic conditions, support for people with high alcohol intake, improved medicines management, and increased uptake of childhood vaccinations. These are all areas that are not well covered through the national incentive scheme (QOF). In addition, there was a specific target to reduce unplanned emergency admissions, which was also not included in the national scheme. Finally, a key component was the involvement of the GPs in developing the scheme, ensuring that they felt the measures were relevant and were collectively committed to the scheme’s success.

As highlighted in Figure 1, these components increased the level of access to GP practices from an average of 50 appointments to 70 appointments per 1,000 needs-weighted population per day. Where GP practices were not achieving the performance indicator targets, they were required to provide evidence outlining actions they were taking to achieve the target. This evidence was reviewed by a committee that included GPs, lay members of the public, contract managers, and representatives from National Health Service (NHS) England. Incentives were in place to achieve the targets, or risk losing income. To date funds have been withdrawn from 37 practices for nonachievement of KPIs over several validation processes. For example, 56 practices had initial submissions that had not met the KPI standards in 2016. Further investigation showed that 35 practices were not able to provide evidence that they met all the standards and this resulted in a £350,000 fund withdrawal from these practices (from nearly £9m invested that year altogether in the scheme). It is therefore hypothesized that LQIS could have had an impact on the level and inequalities of emergency admissions through improvements in the quality of care, increased preventative action (e.g., brief interventions for people with high alcohol intake), early identification of problems, and through addressing unmet needs in previously under resourced practices.

### Data Sources

We used Hospital Episode Statistics provided by NHS Digital along with ONS population estimates to derive our primary outcome: all-cause, all-age emergency hospital admissions per 1,000 population, for all LSOAs from 2005 to 2016. Emergency admissions are defined by NHS digital as those admitted at short notice due to clinical need, generally through an accident and emergency department or through direct request from a GP. Although the KPI used in LQIS was emergency admissions from Ambulatory Care Sensitive Conditions (ACSCs) that are usually managed in primary care, we used the all-cause emergency admission rate as our outcome because previous research suggests that incentive schemes may have an adverse effect on conditions not covered by performance indicators (Damberg et al., 2014; Doran et al. 2008). All-cause emergency admissions would therefore better capture the overall program effect. In sensitivity analysis, we additionally analyzed the impact on emergency admissions for ACSCs. Data on unemployment and demographics was obtained from the ONS. There were no missing data in our study.

### Patient and Public Involvement

This study was part of Partners Priority Program in the National Institute for Health Research Collaboration for Leadership in Applied Health Research and Care North West Coast. The Partners Priority Program aimed to create a strong link between academia and local health care organizations and programs so that a research priority was defined by partners, according to their needs. A coalition of academic researchers, partner organization staff, and public advisors undertook the research. Public advisors were engaged in a qualitative investigation of LQIS that informed the design of this study, in the planning of the work and in the dissemination of the findings.

## Results

The sociodemographic characteristics and emergency admission rates for the intervention populations and the matched control populations before the intervention are summarized in Table 1. Whilst there was some remaining imbalance between the intervention and control populations on the matching variables, these variables were included as covariates in the difference-in-differences model, and any fixed differences between the populations are accounted for through the difference-in-differences analysis.

**Table 1.** Sociodemographic Features of Intervention Population Compared With Unmatched and Matched Populations, in the Time Period Before the Introduction of the Intervention (2005-2010).

	Unmatched sample (2005-2010)			Matched sample (2005-2010)		
Variable	Intervention population, *M* (*SD*)	Unmatched population, *M* (*SD*)	Standardized mean difference	Intervention population *M* (*SD*)	Matched control population, *M* (*SD*)	Standardized mean difference
Average age in years	37.53 (4.39)	39.52 (4.75)	0.435	37.53 (4.39)	38.26 (4.94)	0.155
Working age population unemployed (%)	5.63 (2.89)	2.92 (2.34)	1.032	5.63 (2.89)	3.94 (2.79)	0.595
Population female (%)	50.87 (3.55)	51.06 (2.29)	0.065	50.87 (3.55)	51.18 (2.44)	0.104
All-cause emergency hospital admission rate (per 1,000)	131.52 (43.03)	106.59 (35.37)	0.633	131.52 (43.03)	116.37 (38.36)	0.372
Number of LSOAs	298	4,199	—	298	1,490	—

*Note*. LSOA = Lower Layer Super Output Area. M = mean. SD = standard deviation.

**Figure 2.**
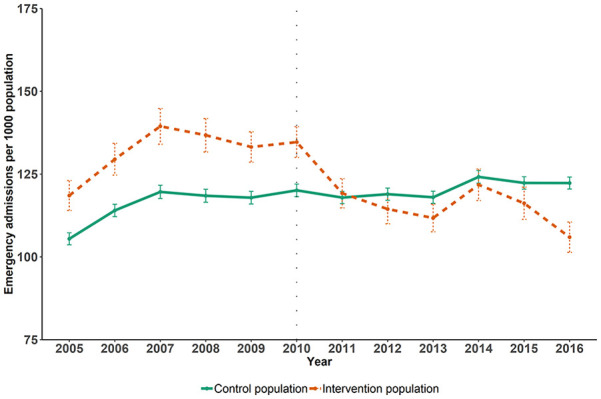
Trends in all-cause emergency hospital admission rates per 1,000 in intervention and control populations, before and after the introduction of the intervention.

Figure 2 shows the trend in emergency admission rates within the intervention and control populations before and after intervention. Whilst the admission rate was higher in the intervention compared with the control populations, prior to the intervention the trends appeared parallel (as suggested by regression analysis in the online supplementary file, Appendix 4). After 2010, when the LQIS was introduced, the emergency admission rate fell in the intervention populations to a similar level as the control populations.

The difference-in-difference analysis (Table 2) shows that there was a statistically significant reduction in admissions in the intervention populations compared with control populations following the introduction of the intervention when controlling for other potential confounders. The analysis indicates that the LQIS was associated with a reduction of 19 emergency admissions per 1,000 people (95% confidence interval [CI: 17, 21) per year, compared with control populations. This is a 14% decline from the baseline level (see Table 1), and approximately equivalent to 10,670 fewer emergency admissions across the overall 550,000 intervention population annually after the intervention, and 64,020 over the 6 years of the intervention period (2011-2016) included in this study. Based on the reference costs for short stay emergency admissions (Curtis & Burns, 2016) used by the NHS, this reduction in admissions is the equivalent to a £39 million cost saving to the NHS over these 6 years.

**Table 2.** Results of DiD Models Showing Changes in All-Cause Emergency Hospital Admissions per 1,000 People in Intervention Populations Following the Introduction of the Intervention, Compared With the Control Populations, 2005-2016.

	Model 1	Model 2	Model 3	Model 4
Variable	Full model, Coefficient [95% CI]	Subgroup: Low deprivation, Coefficient [95% CI]	Subgroup: Middle deprivation, Coefficient [95% CI]	Subgroup: High deprivation, Coefficient [95% CI]
Annual time trend term	1.88 [1.73, 2.04]	1.65 [1.45, 1.85]	1.66 [1.41, 1.90]	1.63 [1.32, 1.95]
Average age in years	1.86 [1.55, 2.17]	2.57 [2.34, 2.81]	2.77 [2.36, 3.17]	3.44 [2.91, 3.97]
Working age population unemployed (%)	1.36 [1.17, 1.55]	1.73 [1.21, 2.25]	1 [0.64, 1.37]	0.89 [0.62, 1.16]
Population female (%)	0.48 [0.10, 0.86]	1.03 [0.64, 1.42]	0.45 [−0.06, 0.95]	−0.36 [–0.99, 0.26]
Group (intervention = 1, control = 0)	14.35 [9.92, 18.79]	14.14 [9.76, 18.52]	28.96 [24.06, 33.85]	15.49 [10.05, 20.93]
Period (postintervention = 1, preintervention = 0)	−7.14 [−8.08, −6.20]	−4.74 [−6.00, −3.48]	−5.2 [−6.72, −3.67]	−10.05 [−11.99, −8.11]
DiD estimator (Group * Period)	−19.4 [−21.34, −17.47]	−17.2 [−19.67, −14.73]	−19.4 [−22.68, −16.11]	−21.6 [–25.34, −17.87]

*Note*. Models include random intercept for LSOA. CIs calculated using cluster robust estimation. Model 1 based on equation shown in the online supplementary file and based on 298 intervention and 1,490 control LSOAs, 21,456 LSOA-years in total. Model 2 based on 100 intervention and 500 control LSOAs, 7,200 LSOA-years in total. Model 3 based on 99 intervention and 495 control LSOAs, 7,128 LSOA-years in total. Model 4 based on 99 intervention and 495 control LSOAs, 7,128 LSOA-years in total. CI = confidence interval; DiD = difference-in-differences; LSOA = Lower Layer Super Output Area.

For each deprivation subgroup, regression analysis suggested parallel trends in the outcome between intervention and control populations before the introduction of the intervention (see online supplementary file, Appendix 5, for details). We found that the effect of the intervention was greater in more deprived areas, with the intervention associated with a decline of 22 emergency admissions per 1,000 population (95% CI [18, 25]) per year in the most deprived third of LSOAs in the study city, compared with a decline of 17 emergency admissions per 1,000 population (95% CI [15, 20]) per year in the least deprived third of LSOAs.

We found similar results when repeating the analysis using GP practices as the units of analysis (decline of 18 emergency admissions per 1,000 population, 95% CI [14, 22] per year), using controls selected from outside the North West region of England (decline of 15 emergency admissions per 1,000 population, 95% CI [13, 17] per year) and using chronic ACSCs as the outcome of interest (decline of 0.8 emergency admissions per 1,000 population, 95% CI [0.7, 0.99] per year; see online supplementary file, Appendix 6).

## Discussion

### Summary

We found that a local General Practice quality improvement scheme that improved the level and equitable distribution of investment alongside a performance incentive scheme was associated with a reduction in emergency hospital admissions. The scheme had a greater impact in more deprived areas compared with more affluent areas, reducing inequalities. This reduction of admissions represented an approximate £39 million cost saving to the NHS. Given that the intervention was estimated to cost £30 million, this suggests that the intervention was cost saving to the NHS.

### Strengths and Limitations

Our study’s strengths include evaluating the LQIS in its real-life implementation setting, which makes our findings potentially more externally valid than those implemented in trials. Our data enabled a follow-up period of 6 years. This allowed us to investigate whether effects were sustained. Third, a combination of quasi-experimental methods (propensity score matching and difference-in-differences) was applied, which can lead to causal estimates of LQIS if the trends in outcomes would have been parallel in the absence of this intervention. Fourth, our reasonably large effective sample size of 21,456 observations provided sufficient power to identify relatively small effects.

Some limitations however are of importance. It is difficult to rule out the possibility that different trends in unobserved confounding factors between the two groups could have influenced the results. Although there were differences between the intervention and control groups, time-invariant differences between the two groups could not bias the results due to the difference-in-differences methods (Angrist & Pischke, 2009). Propensity score matching identified control populations that followed similar trends in the outcome over time prior to the intervention, confirmed by the parallel nature of the trend in emergency admission rates before the intervention. In addition, we controlled for a number of observed confounders. Unobserved confounders therefore could only lead to bias in the results if they followed different time trends over time between the intervention and control groups. We were only able to assess the impact of the intervention on emergency hospital admissions and this may not represent health benefits to the users of these services. Data of other outcomes, such as mortality, was not available at the geographical level required for this analysis and therefore could not be included. The ecological nature of this study limits the conclusions that can be drawn about individual-level factors, and the results represent the population-level impact of the LQIS.

### Comparison With Existing Literature

There are number of reasons that may potentially explain why our findings suggest that LQIS had a positive effect (illustrated in our conceptual framework in Figure 1) whilst the evidence for other similar schemes has been more mixed (Harrison et al., 2014; Ryan et al., 2016; Roland & Guthrie, 2016). First, the LQIS included a significant increase in overall funding for primary care, rather than just reallocating a portion of current funding into a performance incentive scheme, this allowed for increased capacity and access and as well as changes in practice (Doran et al., 2014). Second, the LQIS led to a more equitable distribution of resources between practices relative to need. This could have led to greater benefits by addressing unmet needs in those practices that had previously been under resourced (Doran et al., 2017). Third, the performance metrics were developed to specifically address identified local needs and focused on outcome as well as process measures. Concerns have been raised that although process measures are more easily achievable, they may not lead to the practice change that is needed to improve outcomes (Doran et al., 2017). Fourth, the scheme was developed by GPs and they were involved in its governance, this could have led to better design of performance indicators and greater sense of ownership among the practices, leading to greater changes in practice (Roland & Dudley, 2015; Begum et al., 2013).

### Implications for Practice

Our study has important implications for practice. It indicates that local primary care quality improvement schemes can be effective at reducing demand on secondary care. The study indicates that to be effective they should include increased equitable investment in primary care linked to key outcome based performance measures and involve GPs in their design and governance. Future research should investigate whether these findings are replicated in a trial context investigating overall health benefits and potential adverse effects in areas of care not covered by performance indicators.

## Conclusions

In contrast to other evidence on national incentive schemes, we found that a local scheme developed in collaboration with GPs was effective at reducing rates and inequalities in emergency admissions. Similar approaches could be an effective component of strategies to reduce unplanned hospital admissions elsewhere.

## Supplemental Material

sj-pdf-1-mcr-10.1177_10775587211035280 – Supplemental material for Evaluating the Effectiveness of a Local Primary Care Incentive Scheme: A Difference-in-Differences Study Click here for additional data file. Supplemental material, sj-pdf-1-mcr-10.1177_10775587211035280 for Evaluating the Effectiveness of a Local Primary Care Incentive Scheme: A Difference-in-Differences Study by Esmaeil Khedmati Morasae, Tanith C. Rose, Mark Gabbay, Laura Buckels, Colette Morris, Sharon Poll, Mark Goodall, Rob Barnett and Ben Barr in Medical Care Research and Review
