# Supplementary material for: Evaluating the Effectiveness of a Local Primary Care Incentive Scheme: A Difference-in-Differences Study
Source: Med Care Res Rev. 2021 Jul 29;79(3):394–403. doi: 10.1177/10775587211035280 (PMC9052704; doi:10.1177/10775587211035280)
Supplement: sj-pdf-1-mcr-10.1177_10775587211035280 – Supplemental material for Evaluating the Effectiveness of a Local Primary Care Incentive Scheme: A Difference-in-Differences Study [file sj-pdf-1-mcr-10.1177_10775587211035280.pdf]

Supplementary file

Evaluating the effectiveness of a local primary care incentive scheme: a difference-in-differences study

Contents

- Appendix 1: Outline of the Difference-in-Differences analysis
- Appendix 2: Matching variables
- Appendix 3: Local Quality Improvement Scheme indicators
- Appendix 4: Parallel trends investigation
- Appendix 5: Deprivation subgroup analysis
- Appendix 6: Robustness tests

Appendix 1

Outline of the Difference-in-Differences analysis used in the study:

Difference-in-differences (DiD) analysis is an established approach increasingly used in health research to evaluate the impact of interventions, where the researcher has no role in manipulation of the intervention assignment, sometimes known as "natural experiments"[1,2]. In DiD analyses, outcomes are observed for two groups before and after an intervention and one of the groups is exposed to an intervention in the second period but not in the first period, and the second group is not exposed to the intervention during the study time frame. The average change in outcomes in the second (control) group is then subtracted from the average change in outcomes in the first (intervention) group. This removes biases in second period comparisons between the intervention and control group that could be the result of permanent differences between those groups, as well as biases from comparisons over time in the intervention group that could be the result of trends. Thus the differences-in-differences estimator is therefore:

$$\hat{y} = (\bar{Y}\, Intervention, AFTER - \bar{Y}\, Intervention, Before) - \bar{Y} Comparator, After - \bar{Y}\, Comparator, Before)$$

$\bar{Y}$ is the mean of the outcome variable in the intervention areas after the start of the intervention ($\bar{Y}$ *Intervention*, $AFTER$) and before the start of the intervention ($\bar{Y}$ *Intervention, BEFORE*) and in the comparator areas after the start of the intervention ($\bar{Y}$ Comparator,*After*) and before the start of the intervention ($\bar{Y}$ Comparator,*Before*).

To estimate the DiD parameter, we can run the following regression:

$$Y_{at} = \beta_1 Intervention_a + \beta_2 After_t + \beta_3 After_t * Intervention_a + \varepsilon_{at}$$

where Y_{at} is the outcome variable in area *a* at time *t*, $Intervention_a$ is a dummy variable taking the value 1 for the intervention area and the value 0 for the comparator area and $After_t$ is a dummy variable taking the 1 or the time period after the start of intervention and 0 before. The coefficient of interest is β_3, the coefficient on the interaction term $After_t *$ $Intervention_a$, which is the difference-in-difference parameter.

Since this analysis cannot be biased due to time invariant differences between the intervention and control groups, using a fixed effect model instead of a mixed/random effect model would offer no additional benefits in terms of controlling for unobserved confounding. The DiD analysis could however be biased by trends in other predictors of the outcome if these diverged between treatment and intervention groups after the intervention. Therefore, potential time varying confounders are added to the model to control for such biases.

Equation for multivariable mixed effects linear regression model for emergency hospital admissions:

$$Y_{ij} = \beta_{0j} + \beta_{1j}t_{ij} + \beta_{2j}Age_{ij} + \beta_{3j}Sex_{ij} + \beta_{4j}Unemployment_{ij} + \beta_{5j}Group_{ij} + \beta_{6j}Period_{ij} + \beta_{7j}Group_{ij} * Period_{ij} + \varepsilon_{ij}$$

Where Y_{ij} is the all-cause all-age hospital emergency admission rate per 1000 population for LSOA *i* at year *j*. The intercept (β_{0j}) contains a random effect term and thus varies across years. *t* is an annual time-trend term. Age is the average age of the population in years. Sex is the percent of the population who are female. Unemployment is the percent of the working age population (aged 16–64 years) claiming Jobseeker's Allowance. Group indicates whether LSOA *i* is an intervention or control LSOA (intervention = 1; control = 0). Period indicates whether year *j* occurs post or pre-intervention (post-intervention = 1; pre-intervention = 0). Group*Period is the difference-in-differences estimator.

Appendix 2

Matching variables:

The following variables were included in a propensity score model to match intervention to control populations in the time period before the introduction of the intervention (2005–2010).

Matching variable	Details
Age and gender profile of the population	Annual data on the percentage of the population that were female and the average age of the population per Lower Super Output Area (LSOA) were derived from mid-year population estimates provided by the Office for National Statistics (ONS) (https://www.ons.gov.uk/peoplepopulationandcommunity/populationandmigration/populationestimates/datasets/lowersuperoutputareamidyearpopulationestimates).
Unemployment	Annual unemployment prevalence was calculated using claimant data provided by the ONS. Unemployment was measured as the percentage of people aged 16–64 years claiming Jobseeker's Allowance (https://www.nomisweb.co.uk/).
Emergency admission rate	All-cause, all-age emergency hospital admissions per 1000 population, for all LSOAs from 2005 to 2010. Emergency admissions are defined by NHS digital as those admitted at short notice due to clinical need, generally through an accident and emergency department or through direct request from a general practitioner. Annual emergency admission rates per 1000 population were calculated using Hospital Episode Statistics (HES), with population data obtained from the ONS. Continuous inpatient (CIP) spells were used to calculate emergency admissions per calendar year.

Appendix 3

List of the indicators (KPIs) in the Local Quality Improvement Scheme (LQIS)

No.	KPI	Description
**1**	**Access**	
1.1	Access to General Practice	The average rate per 1000 weighted population of GP / Nurse Practitioner / Clinical pharmacist/ Physician associates/ telephone consultation appointments per week
**2**	**Quality/Prevention**	
2.1	Early Identification	Percentage of registered patients that are included on CHD, HF, Stroke, AF, Hypertension, COPD, and Diabetes registers. Ranges will be measured by standard deviations from the study city Mean
2.1.1		Coronary Heart Disease
2.1.2		Heart Failure
2.1.3		Stroke
2.1.4		Atrial Fibrillation
2.1.5		Hypertension
2.1.6		COPD
2.1.7		Diabetes
2.2	Exception Reporting	Average Exception Reporting Percentage (patient unsuitable and informed dissent only) against register size on the key registers of CHD, HF, Stroke, AF, Hypertension, COPD and Diabetes
2.3	Alcohol	The percentage of patients aged 18 years and over who have had the alcohol consumption recorded in the last 3 years
2.4	Alcohol	The percentage of patients aged 18 years and over who are drinking more than recommended units per week, have their alcohol intake recorded using the AUDIT-C or AUDIT Tool and received a brief intervention in the last 3 years (i.e. those who score 8 and above receive brief advice and those scoring 16 and above are offered specialist support, e.g. referral to LCAS)
2.5	Childhood Vaccinations and Immunisations	The combined percentage achievement (rolling 12 months) for DTaP/IPV/Hib at 1 year, MMR1 at 2 years, PCV booster at 2 years, Hib/MenC booster at 2 years<br>The combined percentage achievement (rolling 12 months) for MMR2 at 5 years and DTaP/IPV preschool booster.
2.6	Palliative/terminal care	Full participation and adherence in the gold standards framework. In order for practices to understand if this is effective then the following measures will be provided to them 1) The % of patients on the palliative care register with preferred place of care recorded on their notes 2) The % of patients on the palliative care register who have died in their preferred place of care
2.7	Dementia	The practice to establish a process to ensure that appropriate Read Codes are included into the patients' medical record upon diagnosis with Mild Cognitive Impairment and to develop an annual recall and review system for people identified with MCI; to review patients with MCI once only at 12 months post diagnosis
**3**	**Use of Resources**	
3.1	ACS admissions	Rate per 1000 hospital weighted population for admissions for a selection of ACS conditions (Angina, Asthma, COPD, Influenza and Pneumonia,) as primary diagnosis.
3.2	Outpatients	Rate per 1000 hospital weighted population for GP referred first Outpatient attendances to certain specialities (Dermatology, ENT, Rheumatology, Urology, Vascular Surgery, Gynaecology)
	**Medicines Management**	
		Patients on warfarin have an INR result in last 4 months
		Patients on lithium should have a lithium level in the last 4 months<br>(Lithium levels should be within a therapeutic range appropriate to the individual patient)
		Patients on lithium should not be prescribed a thiazide
		Patients with dementia should not be prescribed an antipsychotic
		Patients with asthma should not be prescribed a non-cardio specific beta blocker
		Patients with Addison's disease should not be prescribed a thiazide

		Prescribing for type 2 diabetes should avoid risk of hypoglycaemia<br>- T2D on insulin with 2 or more hypo in 12 months<br>- T2D on SU with 2 or more hypo in 12 months<br>Resulting in a hospital admission
**3.4**	Reducing antibiotic use	A target of 5% reduction in weighted volume of antibiotic prescribing against the practice's 2015-16 baseline or achievement of national average volume.
**3.5**	Prescribing of specialist and high cost analgesics	A target of 5% reduction in costs for a combination of pregabalin/oxycodone/buprenorphine patches/fentanyl
4	Improving Quality	
4.1	Significant Event Analysis	Practices with a list size below 3,500 (weighted) to complete 3 clinical significant events using the above format.<br>Practices with a list size above 3,500 (weighted) to complete 5 clinical significant events using the above format.

Appendix 4

Parallel trends test:

Investigating the parallel trends assumption using regression analysis [1]

$$Y_{ij} = \beta_{0j} + \beta_{1j}Age_{ij} + \beta_{2j}Sex_{ij} + \beta_{3j}Unemployment_{ij} + \beta_{4j}Group_{ij} + \beta_{5j}Year_{ij} + \beta_{6j}Group_{ij} * Year_{ij} + \varepsilon_{ij}$$

Where Y_{ij} is the all-cause all-age hospital emergency admission rate per 1000 population for LSOA *i* at year *j* (from 2005 to 2010). The intercept (β_{0j}) is a random effect term and thus varies across years. Age is the average age of the population in years. Sex is the percent of the population who are female. Unemployment is the percent of the working age population (aged 16–64 years) claiming Jobseeker's Allowance. Group indicates whether LSOA *i* is an intervention or control LSOA (intervention = 1; control = 0). Year indicates the year (*j*) pre-intervention. Group*Year is the parallel test estimator.

If the Group*Year parameter is not statistically significant, it suggests that the emergency admissions trends pre-intervention are parallel and the control group can be used in subsequent DiD analyses.

Model outputs:

Result of parallel trend assumption test, only including the pre-intervention data, with an interaction term between intervention group and year (2005-10)

	Coefficient	SE	95% CI	P-value
Average age in years	0.99	0.17	[0.65, 1.33]	< .001
Working age population unemployed (%)	1.51	0.23	[1.06, 1.95]	< .001
Population female (%)	1.24	0.26	[0.72, 1.75]	< .001
Year	1.9	0.15	[1.61, 2.20]	< .001
Group (intervention = 1, control = 0)	13.16	2.44	[8.37, 17.96]	< .001
Parallel Trend estimator (Group*Year)	0.22	0.28	[-0.34, 0.77]	0.441

Model includes random intercept for LSOA
Standard errors and confidence intervals calculated using cluster robust estimation.
Model based on 298 intervention and 1490 control LSOAs, and 10,728 LSOA-years in total
CI = confidence interval; LSOA = Lower-layer Super Output Area

Appendix 5

Deprivation subgroup analysis

Low deprivation subgroup

Socio-demographic features of intervention population compared to unmatched and matched populations, in the time period before the introduction of the intervention (2005 to 2010) for low deprivation subgroup

	**Unmatched sample (2005-2010)**			**Matched sample (2005-2010)**		
	Intervention population	Unmatched population		Intervention population	Matched control population	
	**Mean (SD)**	**Mean (SD)**	**Standardized mean difference**	**Mean (SD)**	**Mean (SD)**	**Standardized mean difference**
Average age in years	37.54 (6.12)	41.74 (4.56)	0.779	37.54 (6.12)	40.43 (4.87)	0.523
Working age population unemployed (%)	2.87 (1.25)	1.24 (1.02)	1.427	2.87 (1.25)	1.61 (1.38)	0.958
Population female (%)	50.41 (3.36)	50.71 (2.30)	0.105	50.41 (3.36)	50.91 (2.41)	0.171
All-cause emergency hospital admission rate (per 1000)	93.37 (28.38)	78.56 (19.61)	0.607	93.37 (28.38)	84.99 (21.28)	0.334
Number of LSOAs	100	1314	-	100	500	-
LSOA = Lower-layer Super Output Area; SD = standard deviation						

Result of parallel trend assumption test, only including the pre-intervention data, with an interaction term between intervention group and year (2005-10) for low deprivation subgroup

	Coefficient	SE	95% CI	P-value
Average age in years	2.53	0.13	[2.28, 2.79]	< .001
Working age population unemployed (%)	0.32	0.46	[-0.59, 1.23]	0.489
Population female (%)	0.41	0.25	[-0.08, 0.89]	0.103
Year	2.09	0.19	[1.72, 2.45]	< .001
Group (intervention = 1, control = 0)	15.18	2.59	[10.10, 20.27]	< .001
Parallel Trend estimator (Group*Year)	0.13	0.43	[-0.72, 0.97]	0.764

Model includes random intercept for LSOA
Standard errors and confidence intervals calculated using cluster robust estimation.
Model based on 100 intervention and 500 control LSOAs, and 3600 LSOA-years in total
CI = confidence interval; LSOA = Lower-layer Super Output Area

Middle deprivation subgroup

Socio-demographic features of intervention population compared to unmatched and matched populations, in the time period before the introduction of the intervention (2005 to 2010) for middle deprivation subgroup

	Unmatched sample (2005-2010)			Matched sample (2005-2010)		
	Intervention population	Unmatched population		Intervention population	Matched control population	
	**Mean (SD)**	**Mean (SD)**	**Standardized mean difference**	**Mean (SD)**	**Mean (SD)**	**Standardized mean difference**
Average age in years	38.19 (3.19)	40.53 (4.21)	0.625	38.19 (3.19)	39.42 (4.10)	0.334
Working age population unemployed (%)	5.67 (1.87)	2.10 (1.10)	2.326	5.67 (1.87)	2.56 (1.18)	1.991
Population female (%)	50.53 (3.65)	51.20 (2.14)	0.225	50.53 (3.65)	51.25 (2.12)	0.244
All-cause emergency hospital admission rate (per 1000)	135.06 (30.19)	99.31 (23.37)	1.324	135.06 (30.19)	106.70 (24.62)	1.03
Number of LSOAs	99	1176	-	99	495	-
LSOA = Lower-layer Super Output Area; SD = standard deviation						

Result of parallel trend assumption test, only including the pre-intervention data, with an interaction term between intervention group and year (2005-10) for middle deprivation subgroup

	Coefficient	SE	95% CI	P-value
Average age in years	2.72	0.2	[2.33, 3.11]	< .001
Working age population unemployed (%)	-0.08	0.34	[-0.75, 0.59]	0.815
Population female (%)	0.43	0.28	[-0.12, 0.97]	0.123
Year	2.24	0.22	[1.81, 2.68]	< .001
Group (intervention = 1, control = 0)	32.19	3.09	[26.13, 38.25]	< .001
Parallel Trend estimator (Group*Year)	0.03	0.44	[-0.84, 0.90]	0.95

Model includes random intercept for LSOA
Standard errors and confidence intervals calculated using cluster robust estimation.
Model based on 99 intervention and 495 control LSOAs, and 3564 LSOA-years in total
CI = confidence interval; LSOA = Lower-layer Super Output Area

High deprivation subgroup

Socio-demographic features of intervention population compared to unmatched and matched populations, in the time period before the introduction of the intervention (2005 to 2010) for high deprivation subgroup

	**Unmatched sample (2005-2010)**			**Matched sample (2005-2010)**		
	Intervention population	Unmatched population		Intervention population	Matched control population	
	**Mean (SD)**	**Mean (SD)**	**Standardized mean difference**	**Mean (SD)**	**Mean (SD)**	**Standardized mean difference**
Average age in years	36.86 (3.03)	37.12 (4.12)	0.071	36.86 (3.03)	36.77 (4.40)	0.023
Working age population unemployed (%)	8.39 (2.19)	4.78 (2.38)	1.578	8.39 (2.19)	6.54 (2.59)	0.773
Population female (%)	51.67 (3.50)	51.23 (2.36)	0.147	51.67 (3.50)	51.55 (2.96)	0.037
All-cause emergency hospital admission rate (per 1000)	166.51 (33.83)	133.16 (32.50)	1.005	166.51 (33.83)	149.11 (33.98)	0.513
Number of LSOAs	99	1709	-	99	495	-
LSOA = Lower-layer Super Output Area; SD = standard deviation						

Result of parallel trend assumption test, only including the pre-intervention data, with an interaction term between intervention group and year (2005-10) for high deprivation subgroup

	Coefficient	SE	95% CI	P-value
Average age in years	3.05	0.29	[2.48, 3.63]	< .001
Working age population unemployed (%)	0.72	0.27	[0.20, 1.24]	0.007
Population female (%)	0.66	0.3	[0.07, 1.25]	0.028
Year	1.75	0.27	[1.23, 2.27]	< .001
Group (intervention = 1, control = 0)	14.35	3.16	[8.16, 20.55]	< .001
Parallel Trend estimator (Group*Year)	0.55	0.57	[-0.57, 1.66]	0.338

Model includes random intercept for LSOA
Standard errors and confidence intervals calculated using cluster robust estimation.
Model based on 99 intervention and 495 control LSOAs, and 3564 LSOA-years in total
CI = confidence interval; LSOA = Lower-layer Super Output Area

Appendix 6

Robustness tests

GP practice-years as the units of analysis

The DiD analysis using GP practice-years as the units of the analysis showed that the LQIS was statistically significantly associated with reduction of all-cause emergency admissions in the intervention practices compared to the control practices, supporting the primary findings at LSOA-year level.

Features of intervention GP practices compared to unmatched and matched practices, in the time period before the introduction of the intervention (2005 to 2010)

	Unmatched sample (2005-2010)			Matched sample (2005-2010)		
	Intervention practices	Unmatched practices		Intervention practices	Matched control practices	
	Mean (SD)	Mean (SD)	Standardized mean difference	Mean (SD)	Mean (SD)	Standardized mean difference
Average age in years	37.84 (2.36)	39.12 (3.21)	0.455	37.84 (2.36)	39.31 (3.89)	0.457
Working age population unemployed (%)	5.93 (1.99)	3.08 (1.67)	1.547	5.93 (1.99)	3.37 (1.90)	1.313
Population female (%)	51.20 (1.42)	51.04 (1.02)	0.13	51.20 (1.42)	51.13 (1.24)	0.052
All-cause emergency hospital admission rate (per 1000)	125.85 (32.57)	101.03 (25.27)	0.852	125.85 (32.57)	102.48 (27.22)	0.778
Practice size (patients)	5073.98 (3165.52)	6172.94 (3545.45)	0.327	5073.98 (3165.52)	5960.91 (3578.55)	0.263
Number of practices	92	1008	-	92	460	-
GP = General practitioner; SD = standard deviation						

Result of parallel trend assumption test, only including the pre-intervention data, with an interaction term between intervention group and year (2005-10)

	Coefficient	SE	95% CI	P-value
Average age in years	-0.6	0.38	[-1.35, 0.15]	0.12
Working age population unemployed (%)	3.52	0.49	[2.57, 4.47]	< .001
Population female (%)	4.74	1.47	[1.85, 7.64]	0.001
Year	0.87	0.31	[0.26, 1.48]	0.005
Group (intervention = 1, control = 0)	11.18	3.64	[4.04, 18.33]	0.002
Parallel Trend estimator (Group*Year)	0.8	0.57	[-0.32, 1.92]	0.163

Model includes random intercept for GP practices
Standard errors and confidence intervals calculated using cluster robust estimation.
Model based on 92 intervention GP practices and 460 control GP practices, and 3,312 practice-years in total
CI = confidence interval; GP = General Practitioners

Result of difference-in-differences analysis showing the change in all-cause emergency hospital admissions per 1000 in intervention GP practices following the introduction of the intervention, compared to the control practices, 2005-2016

	Coefficient	SE	95% CI	P-value
Annual time trend term	1.78	0.14	[1.50, 2.05]	< .001
Average age in years	0.24	0.28	[-0.31, 0.80]	0.391
Working age population unemployed (%)	1.64	0.19	[1.26, 2.02]	< .001
Population female (%)	1.85	1.07	[-0.26, 3.95]	0.085
Group (intervention = 1, control = 0)	19.41	3.02	[13.48, 25.34]	< .001
Period (post-intervention =1, pre-intervention=0)	-3.07	0.76	[-4.55, -1.58]	< .001
DiD estimator (Group*Period)	-18.23	2.05	[-22.25, -14.22]	< .001

Model includes random intercept for GP practices
Standard errors and confidence intervals calculated using cluster robust estimation.
Model based on 92 intervention GP practices and 460 control GP practices, and 6,624 practice-years in total
CI = confidence interval; DiD = Difference-in-Differences; GP = General Practitioners

Emergency hospital admissions due to chronic Ambulatory Care Sensitive Conditions (ACSCs) as the outcome of interest

The main analysis was repeated using an alternative outcome: emergency hospital admissions due to chronic ACSCs. The ICD-10 codes included in the definition of chronic ACSCs were: J45, J46, E10-E14.

The DiD analysis indicates that the LQIS was statistically significantly associated with a reduction in emergency admissions due to chronic ACSCs in the intervention compared to the control populations.

Result of parallel trend assumption test, only including the pre-intervention data, with an interaction term between intervention group and year (2005-10)

	Coefficient	SE	95% CI	P-value
Average age in years	-0.07	0.01	[-0.09, -0.06]	< .001
Working age population unemployed (%)	0.16	0.01	[0.14, 0.18]	< .001
Population female (%)	0.11	0.01	[0.08, 0.14]	< .001
Year	0.02	0.01	[0.00, 0.05]	0.097
Group (intervention = 1, control = 0)	0.24	0.12	[0.00, 0.47]	0.051
Parallel Trend estimator (Group*Year)	-0.02	0.03	[-0.09, 0.04]	0.451

Model includes random intercept for LSOA
Standard errors and confidence intervals calculated using cluster robust estimation.
Model based on 298 intervention and 1490 control LSOAs, and 10,728 LSOA-years in total
CI = confidence interval; LSOA = Lower-layer Super Output Area; SE = standard error

Result of difference-in-differences analysis showing the change in emergency hospital admissions due to chronic ACSCs per 1000 people in the intervention population following the introduction of the intervention relative to the control populations, 2005-16

	Coefficient	SE	95% CI	P-value
Annual time trend term	0.09	0.01	[0.07, 0.10]	< .001
Average age in years	-0.09	0.01	[-0.10, -0.08]	< .001
Working age population unemployed (%)	0.08	0.01	[0.06, 0.09]	< .001
Population female (%)	0.07	0.01	[0.05, 0.09]	< .001
Group (intervention = 1, control = 0)	0.28	0.09	[0.10, 0.46]	0.003
Period (post-intervention =1, pre-intervention=0)	-0.44	0.05	[-0.54, -0.33]	< .001
DiD estimator (Group*Period)	-0.83	0.09	[-0.99, -0.66]	< .001

Model includes random intercept for LSOA
Standard errors and confidence intervals calculated using cluster robust estimation.
Model based on 298 study city and 1490 control LSOAs, and 21,456 LSOA-years in total
ACSC = Ambulatory Care Sensitive Conditions; CI = confidence interval; DiD = Difference-in-Differences; LSOA = Lower-layer Super Output Area

Analysis using controls selected from outside the North West region of England

Result of parallel trend assumption test, only including the pre-intervention data, with an interaction term between intervention group and year (2005-10)

	Coefficient	SE	95% CI	P-value
Average age in years	1.85	0.21	[1.44, 2.26]	< .001
Working age population unemployed (%)	-0.08	0.33	[-0.73, 0.58]	0.821
Population female (%)	0.8	0.36	[0.09, 1.52]	0.027
Year	2.57	0.21	[2.16, 2.98]	< .001
Group (intervention = 1, control = 0)	8.49	2.51	[3.58, 13.41]	< .001
Parallel Trend estimator (Group*Year)	-0.04	0.29	[-0.62, 0.54]	0.891

Model includes random intercept for LSOA
Standard errors and confidence intervals calculated using cluster robust estimation.
Model based on 298 intervention and 1490 control LSOAs, and 10,728 LSOA-years in total
CI = confidence interval; LSOA = Lower-layer Super Output Area

Result of difference-in-differences analysis showing the change in all-cause emergency hospital admissions per 1000 people in intervention populations following the introduction of the intervention relative to the control populations, 2005-16

	Coefficient	SE	95% CI	P-value
Annual time trend term	1.16	0.15	[0.88, 1.45]	< .001
Average age in years	2.78	0.26	[2.26, 3.30]	< .001
Working age population unemployed (%)	0.73	0.49	[-0.22, 1.68]	0.133
Population female (%)	0.75	0.25	[0.27, 1.23]	0.002
Group (intervention = 1, control = 0)	7.73	2.31	[3.19, 12.27]	< .001
Period (post-intervention =1, pre-intervention=0)	-8.37	0.89	[-10.12, -6.62]	< .001
DiD estimator (Group*Period)	-15.08	1.12	[-17.27, -12.89]	< .001

Model includes random intercept for LSOA
Standard errors and confidence intervals calculated using cluster robust estimation.
Model based on 298 study city and 1490 control LSOAs, and 21,456 LSOA-years in total
CI = confidence interval; DiD = Difference-in-Differences; LSOA = Lower-layer Super Output Area

Analysis applying the synthetic control method

As matching can introduce bias related to regression to the mean,[3] we conducted additional analysis that includes a weighted combination of all of the available "untreated" units, instead of just a matched sample. In this analysis we apply the synthetic control method for microdata developed by Robbins et al. to estimate the intervention effect.[4,5] The synthetic control method is a generalisation of difference-in-differences methods, whereby an untreated version of the treated cases (i.e. a synthetic control) is created using a weighted combination of untreated cases.

To construct the synthetic control group, we derive calibration weights such that the weighted characteristics of the control LSOAs population (emergency admissions, age, unemployment, proportion of the population that were female population) match those of the intervention population in the period before the intervention (2005-2010) The weighting algorithm derives weights that meet three constraints. Firstly, the sum of weights in the control group equals the number of emergency admissions in the intervention group. Secondly, the weighted average of each of the characteristics above in the synthetic control group matches those in the intervention group. Lastly, the synthetic control and intervention group also match across all pre-intervention time points in terms of the numbers of admissions.[4]

The Average Treatment Effect for the Treated (ATT) is estimated as the difference in cumulative number of admissions in the intervention group period after the intervention time point (2011-2016), compared to the (weighted) number of admissions in the synthetic control group. To estimate the 95% confidence intervals and p-values we apply a permutation procedure, through repeating the analysis through 250 placebo permutations randomly allocating the intervention LSOAs to the intervention group, to estimate the sampling distribution of the treatment effect and calculating permuted p-values and confidence intervals.[5] All analysis was performed using the Microsynth package.[4]

The figure below shows the trend in the emergency admission rate in the intervention and synthetic control populations. Similarly to our main matched difference-in-differences analysis, in the intervention population the rate of admissions falls relative to the synthetic control group following the intervention. The effect size from this analysis was similar to our main analysis – indicating that the intervention was associated with a 12% decline in admissions (95% CI 11% to 14%).

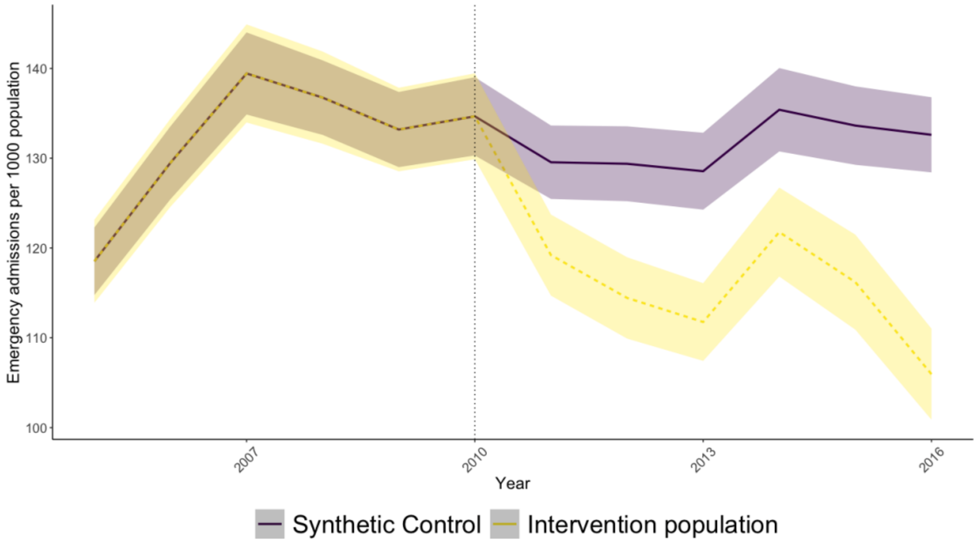

References:

1 Craig P, Katikireddi SV, Leyland A, Popham F. Natural Experiments: An Overview of Methods, Approaches, and Contributions to Public Health Intervention Research. *Annu Rev Public Health* 2017; **38**: 39–56.

2 Wing C, Simon K, Bello-Gomez RA. Designing Difference in Difference Studies: Best Practices for Public Health Policy Research. *Annual Review of Public Health* 2018; **39**: 453–69.

3 Daw JR, Hatfield LA. Matching and Regression to the Mean in Difference-in-Differences Analysis. *Health Services Research* 2018; **53**: 4138–56.

4 Robbins MW, Davenport S. Microsynth: Synthetic Control Methods for Disaggregated and Micro-Level Data in R. *Journal of Statistical Software; Vol 1, Issue 2 (2021)* 2021; published online Jan 14. https://www.jstatsoft.org/v097/i02.

5 Robbins MW, Saunders J, Kilmer B. A Framework for Synthetic Control Methods With High-Dimensional, Micro-Level Data: Evaluating a Neighborhood-Specific Crime Intervention. *Journal of the American Statistical Association* 2017; **112**: 109–26.
